# Nanomaterials for Protein Delivery in Anticancer Applications

**DOI:** 10.3390/pharmaceutics13020155

**Published:** 2021-01-25

**Authors:** Anne Yau, Jinhyung Lee, Yupeng Chen

**Affiliations:** Department of Biomedical Engineering, University of Connecticut, Storrs, CT 06269, USA; anne.yau@uconn.edu (A.Y.); jinhyung.lee@uconn.edu (J.L.)

**Keywords:** nanomaterials, protein delivery, anticancer therapy, cancer treatment, nanotechnology

## Abstract

Nanotechnology platforms, such as nanoparticles, liposomes, dendrimers, and micelles have been studied extensively for various drug deliveries, to treat or prevent diseases by modulating physiological or pathological processes. The delivery drug molecules range from traditional small molecules to recently developed biologics, such as proteins, peptides, and nucleic acids. Among them, proteins have shown a series of advantages and potential in various therapeutic applications, such as introducing therapeutic proteins due to genetic defects, or used as nanocarriers for anticancer agents to decelerate tumor growth or control metastasis. This review discusses the existing nanoparticle delivery systems, introducing design strategies, advantages of using each system, and possible limitations. Moreover, we will examine the intracellular delivery of different protein therapeutics, such as antibodies, antigens, and gene editing proteins into the host cells to achieve anticancer effects and cancer vaccines. Finally, we explore the current applications of protein delivery in anticancer treatments.

## 1. Introduction

Advances made in the nanotechnology field have opened different possibilities in medical sciences, such as the field of drug delivery. Various biomaterials are developed as drug delivery carriers to overcome challenges, such as low efficiency in targeting specific tissue and cells, or short half-life in circulation [1,2]. One such example used as a drug delivery platform is nanoparticles. Nanoparticles are used as drug and protein delivery platforms and have gained attention due to their ability to overcome different challenges faced by the current delivery platforms. Many nanoparticles offer advantages with high bioactivity efficacy, less side effects for healthy tissues, long-acting time with controllable release, and improved intracellular penetration [3,4,5].

While many chemotherapeutic agents used to treat cancers often come with serious side effects due to their toxicity, various researchers have started to search for more effective alternatives to decrease the toxicity of anticancer agents and to enhance selective uptake in tumor tissue, including the increase of half-life of anticancer agents. The use of nanoparticles as delivery vehicles is one way to overcome the challenges faced by protein or peptide agents. Antibodies, immunostimulatory agents, and Cas9 proteins used in cancer treatments have the inability to penetrate cell efficiently and reach the cytosols due to their hydrodynamic sizes and surface chemistry. For example, the antibodies’ nonspecific interactions with protease, nucleases, and immune cells might reduce efficacy and cause adverse events, as well as endosomal entrapment and degradation of the cargoes that could impede the successful delivery into cells. Therefore, targeting diseased cells is crucial to avoid adverse effects in healthy cells or tissues. Improving the efficacy of treatments by modifying cancer treatments is highly attractive [6,7,8]. This review covers the types of nanoparticles typically used as drug delivery platforms, as well as the current applications of protein delivery, such as vaccines, targeted delivery, and immunotherapy in anticancer treatments.

## 2. Types of Nanoparticles

**Figure 1:**
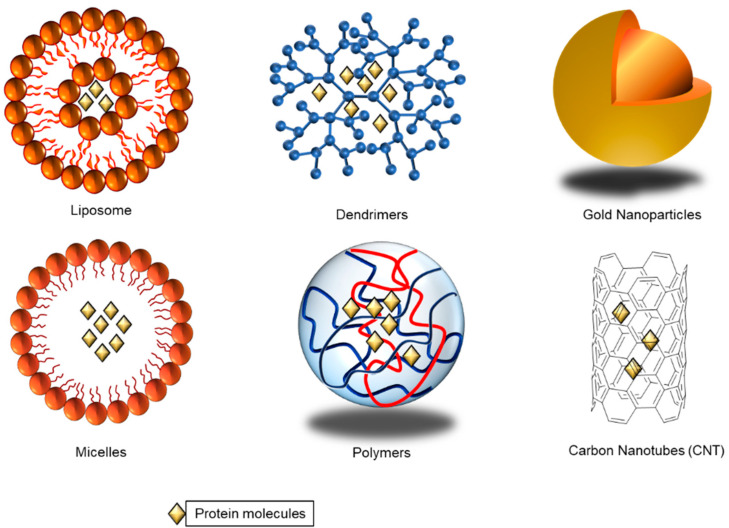
Types of nanoparticles used to carry drug or protein delivery into cells.

The nano delivery system offers several benefits, including protecting cargoes, prolonging circulation half-life, higher cellular uptake, and enhancing the payload’s biological stability. Approaches, such as viral vector and genetic engineering, have studied extensively in vitro and in vivo assays, and have shown limited success due to their advantages and limitations [7]. Various particles, such as lipid nanoparticles, and biomimetic nanoparticles, such as biopolymer-based nanoparticles, and DNA-based nanoparticles, as seen in Figure 1, have been explored as drug delivery platforms to overcome the mentioned issues. Different factors were considered when selecting nanoparticles for different drugs and proteins as delivery carriers. Encapsulation of anticancer drugs in a lipid layer can help its uptake into cells without any side effects. Once in the cell, the lipid layer will be broken down efficiently releasing the encapsulated drugs inside [8]. In addition to using nanoparticles, the newly discovered genome editing tool, the Clustered Regularly Interspaced Short Palindromic Repeats with CRIPSR-associated nuclease-9 (CRISPR/Cas9) has shown unprecedented clinical potential to target cancer diseases due to its high accuracy and efficiency, which is covered in this review.

The size, shape, and surface properties of nanoparticles could affect the ability of nanotherapeutic platforms. Nanoparticles are solid colloidal particles ranging between 10 and 1000 nm. One of the major advantages of using nanoparticles as a drug or protein delivery platform is the capability of manipulating particle size and surface properties to achieve different site-specific actions of drugs at an optimal rate and dosage [9]. Not only can the particle size, morphology, and surface charge be controlled, nanoparticles also offer the ability to carry or deliver different therapeutic or diagnostic agents, such as small molecules or proteins while releasing active molecules in a controlled manner [9]. With the improved surface properties, it can also improve the solubility and stability of encapsulated drugs, increasing the change of screening drugs that were previously ignored due to poor pharmacokinetics.

Monoclonal antibodies can be considered a type of protein-based drug where the antibodies are engineered to target cell death (programmed death receptor 1, PD-1) or cytotoxic T-lymphocyte-associated antigen 4 (CTLA-4) and are commercialized to be used in the clinic to treat different cancers [10]. Polymeric nanoparticles and hydrogel nanoparticles are becoming more attractive because they possess special characteristics that other nanoparticles do not have, such as good biocompatibility due to their high water content and highly tunable properties. Many hydrogels can be synthesized through crosslinking approaches making them more robust, tough, and elastic, while the size of the polymer nanoparticles can be easily controlled [11]. The increased interest in the use of injectable, biomimetic, and biocompatible nanoparticles for anticancer drug delivery has led to the development of Janus-based nanotubes (JBNTs) that have demonstrated to be effective in regenerative medicine due to their biologically derived structure and chemical properties [12]. Deriving from the fundamental and biological building block found in living organisms, DNA is important because it contains hereditary materials and genes. The non-covalent Janus base-nanomaterials have shown better biocompatibility and biodegradability compared to conventional drug delivery vehicles.

Protein-based drugs have emerged to be one of the most promising candidates for treatments of different diseases, especially for cancer treatment, due to their high potency and specificity in anticancer effects by either directly inducing cancer cell apoptosis or inhibiting tumors with manipulation of the microenvironment [13,14]. Even at low concentrations, protein-based drugs have shown high specific activity when compared with small-molecule drugs [15]. Due to the astounding development of therapeutic antibodies and the convenience it affords, researchers also started looking into high concentration protein solutions to be injected subcutaneously [16]. Mutation of normal cells to cancer typically leads to the expression of neoantigens, which can be detected by the immune system. Certain cancer cells seem to be able to escape detection resulting in the development of cancer treatment to boost the immune system. For example, checkpoint-inhibiting monoclonal antibodies (mAbs) are immune system proteins made in the laboratory designed to recognize and bind to other unique proteins in the body. While antibodies are produced naturally by the body and help in detecting and destroying foreign bodies, the assistance of monoclonal antibodies enhances the recognition of specific targets of specific receptors found in tumor cells. The amalgamation of nanotechnology in cancer research has vastly improved the development research areas, such as increasing the half-life of drugs or regulating the immune system more efficient in combating cancer [17,18,19,20].

### 2.1. Lipid Nanoparticles

Lipid nanoparticles have been greatly utilized for cancer therapy and drug delivery. For instance, researchers in the Department of Chemistry at Ludwig Maximilian University of Munich (LMU) developed a class of novel amorphous nanoparticles consisting of citrate and calcium phosphate, capable of passing the cellular barriers during the uptake and killing tumor cells in a more targeted approach [21,22]. The main components of the lipid nanoparticles are phospholipids, which are organized in a bilayer structure due to their amphipathic properties. In an aqueous environment, they aligned themselves, forming vesicles, improving solubility, and stability of any anticancer drugs once they are loaded into the particles. Furthermore, they can be controlled to encapsulate either hydrophobic or hydrophilic drugs by the addition of other components, such as cholesterol or other compounds during the fabrication of the lipid nanoparticles [23]. Extensive studies have been investigated in the development of new liposomes. For instance, Yan et al. [24] developed a pH-responsive liposome with a terminal amine group coated with glycol derivative chitosan. Doxorubicin (DOX) was loaded, and an increase of anticancer efficacy was observed in their study. On the other hand, liposomes can be developed to be multi-purposed, where they could be used as both anticancer and imaging probes, at the same time, when DOX is encapsulated together with Magnevist^®^, a contrasting agent in a liposome modified with amphiphilic hyaluronic acid and cholesterol [25].

Lipid nanoparticles have been considered the most promising intracellular antibody delivery, providing high in vivo efficiency and stability of the cargo and biocompatibility. However, the high molecular weight of antibodies may show low effective encapsulation; therefore, requiring additional steps for encapsulation causing leakage of antibody issues, and modification of endosomal escape [26]. Several cationic lipid-based nanoparticles have been proposed for the intracellular delivery of antibodies. Behr et al. reported di-octadecyl-amido-glycyl-spermine (DOGS) lipid structure for the intracellular delivery of antibodies. DOGS lipid nanoparticles have shown successful transfection of anti-alpha-tubulin and anti-beta-actin antibodies to cytoplasmic, leading to cytoskeleton fiber depolymerization [27]. Courtete et al. demonstrated that lipid nanoparticles can deliver anti-HPV16 E6 antibody and resulted in the downregulation of this oncoprotein activity in vivo [28]. Another example is solid lipid nanoparticles (SLN), Battaglia et al. produced bevacizumab loaded SLN and successfully delivered bevacizumab with increased activity of 100–200-fold, in vitro [29]. Recently, the liposomal carrier has successfully delivered immunostimulants to enhance their immune activation properties. Zhang Y et al. reported PEGylated liposomes could deliver interleukin (IL)-2 and anti-CD137 (cluster of differentiation) mAb to the tumor and effectively delay tumor growth [30]. A wide range of lipid nanoparticles has been investigated to deliver CRISPR/Cas9 systems. One example is lipid–gold nanoparticle formulation, which was surface modified with an HIV-1 Tat peptide for nuclear targeting and complexed with the Cas9 protein and pDNA encoding sgRNA targeting Plk1 of the human malignant melanoma cell line [31]. Recently, lipid-like nanoparticles for CRISPR/Cas9 RNP delivery showed promising results. Lipid-like nanoparticles are made of lipidoid, which is composed of phospholipids, cholesterol, and polyethylene glycol. Lipidoid NPs were loaded with CRISPR/Cas9 RNP and could deliver RNP to HEK293-Green Fluorescence Protein (GFP) cells that demonstrate 60% GFP expression knockdown [32]. The constant developments of lipid nanoparticles have led to the development of solid lipid nanoparticles and nanostructured lipid carriers, which are covered briefly in this review.

### 2.2. Viral Vectors

Oncolytic viruses (OVs) are emerging vectors in cancer therapeutics. These viruses were genetically modified to lack virulence to the normal cells, but maintain the capacity to attack and lyse cancer cells [33]. One of the strategies for targeting OVs is using tumor-specific promoters, viral gene knockout, or capsid modification. The other is to load the virus with immune system-activating agents, such as cytokines, antibodies, and costimulatory molecules to reverse the immunosuppressive tumor microenvironment [2]. For example, a range of viruses has been investigated to load with anti-VEGF (vascular endothelial growth factor) single-chain antibodies to inhibit tumor neovascularization [34]. Nevertheless, despite extensive research, oncolytic virotherapy has shown limited efficacy against solid tumors due to the physical barriers of the cell’s endothelial and dense extracellular matrix (ECM) [35].

Most viral vectors are limited to their load size, such as large protein, including Cas9 protein. Therefore, alternatively, virus-like particles (VLPs) derived from a modified version of the viral genome can deliver a wide range of protein sizes ranging from peptides to large proteins to cells. A protein of interest can be appended with targeting peptides, resulting in their efficient incorporation into VLPs. Stanislaw J. Kaczmarczyk et al. showed intracellular delivery of proteins using VLP that can deliver proteins to either the surface or the cells’ intracellular [36]. Recently, nanoblades have been designed based on the murine leukemia virus (MLV) that can successfully transfer Cas9–sgRNA ribonucleoproteins (RNP)s to cell line in vitro and in vivo [37].

### 2.3. Polymeric Nanocarriers

Polymer nanoparticles are extensively applied as biomaterials for drug delivery. They show remarkable enhancement over intravenous and oral administration routes and transport active cargoes to targeted tissues or organs with controlled release. Therefore, polymeric nanoparticles are considered to be ideal candidates for cancer therapy due to their capacity to adjust drug release. [37]. Polymeric nanoparticles can be formulated by either natural or synthetic monomers. The antibody can be embedded in the nanoparticle surface, entrapped, or dispersed throughout the matrix. The synthetic polymer made with poly (lactic-co-glycolic acid) (PLGA) is one of the most used polymers for antibody delivery due to the biocompatibilities and biodegradability. In addition, there is a benefit, which antibody release of PLGA nanocarrier can be controlled. Cationic polymeric vectors can achieve tumor cell targeting ability and deliver antibodies [38]. Varshocian et al. reported that the release of encapsulated bevacizumab in the PLGA nanoparticles had been delayed [39]. A natural polymer, such as chitosan nanoparticles, exhibit antitumor activity by membrane disruption and induce apoptosis. Therefore, chitosan nanoparticles as carriers for anticancer antibodies may show a synergistic effect for anticancer therapy. The encapsulating antibody can be prepared by ionic gelation. Pan et al. showed the formulation of antibody-loaded chitosan nanoparticles by changing the pH of the system [40]. Gdowski et al. reported encapsulation of anti-Annexin A2 (anti-Anx A2) into PLGA nanoparticles, but low efficiency of encapsulation [41].

Polymeric nanoparticles have been widely used for the delivery of immunostimulatory agents. For instance, PEG-PLGA nanoparticles encapsulate the tumor antigen (Imiquimod, R837) and deliver to the immune cells, priming the site for immune activity [38]. Another example is the delivery of anti-OX40 mAbs, in which OX40 is an important tumor necrosis factor (TNF) receptor on the surface of activated immune cells and regulates the CD4 + and CD8 + T cells. The anti-OX40 mAbs were attached to PLGA nanoparticles by conjugation onto the surface and promoted increased proliferation and activation of CTLs in vitro [39].

Several groups have reported that a polymeric vector-based CRISPR/Cas9 system targeting proto-oncogenes could disrupt the target gene and suppress tumor growth. Polyethylenimine (PEI) was one of the several polymers first applied to deliver Cas9 protein. Choung et al. has covalently linked the Cas9 to the PEI, which successfully showed endosomal escape and released the Cas9 protein delivery [40]. Recently, Gong et al. designed a series of redox responsive copolymer for delivery of Cas9 ribonucleoprotein. The redox-responsive disulfide bond makes the redox-responsiveness release the payloads rapidly in the cytosol, where the GSH level is relatively high [41].

### 2.4. Dendrimer Nanoparticles

Dendrimers can also be applied to a variety of cancer treatments to improve their efficacy and safety. The dendrimer nanocarriers have been demonstrated to decrease non-specific toxicities, enhance drug stability and bioavailability, improve drug delivery profiles, and target drug delivery. However, the dendrimer’s synthesis is laborious, and the balance between biodegradability and toxicity highly relies on the scaffold [42].

In protein delivery, dendrimers need to form stable nanostructures with proteins to protect them from enzymatic degradation and deliver them into cells. One example is Polyamidoamine (PAMAM) dendrons, with a salicyl hydroxamate core attached to the boronic acid-modified proteins, successfully delivering attached protein drugs into the cancer cells [43]. Recently, Jia Lv et al. developed a bifunctional and bio-reducible dendrimer bearing a fluoroalkyl tail for efficient cytosolic protein delivery. The polymer PBA-rich fluorinated dendrimer (PFD) delivered a therapeutic toxin protein into cancer cells and successfully retarded the tumor growth in 4T1 tumor-bearing mice [44]. To deliver Cas9 protein, Chongyi Liu et al. reported that boronic acid-rich dendrimers can efficiently deliver native proteins. The negative dendrimers encapsulated the positively charged proteins and efficiently delivered the Cas9 protein, which led to high efficiency in CRISPR-Cas9 genome editing [45].

### 2.5. Biomimetic Nanoparticles

Biomimetic nanomaterials have good biocompatibility and interesting chemical properties. Cell-penetrating peptides (CPPs) were used for intracellular antibody delivery, having demonstrated their ability to cross the cellular membrane. Until now, CPPs have been linked to proteins; covalent linkage or non-covalent linkage have been applied [46]. For instance, arginine-rich CPPs, including Human immunodeficiency virus type -1 (HIB-1) TAT-derived peptides with full-length mAb heavy chains were directed against the multi-functional HBx protein of the hepatitis B virus (HBV) into an infected human hepatic Huh7 cell line. It was reported that an arginine-rich (R9) CPP was conjugated to an anti-HCV-scFV to target the hepatitis C virus (HCV), leading to inhibition of HCV replication in an infected Huh7 cell line [47]. Although CPP conjugated with antibodies shows promising results in vitro due to efficient endosomal escape, high biocompatibility, and low cytotoxicity, challenges involve targeting in vivo due to the lack of specific cell targeting and low internalization efficiency [46].

**Figure 2:**
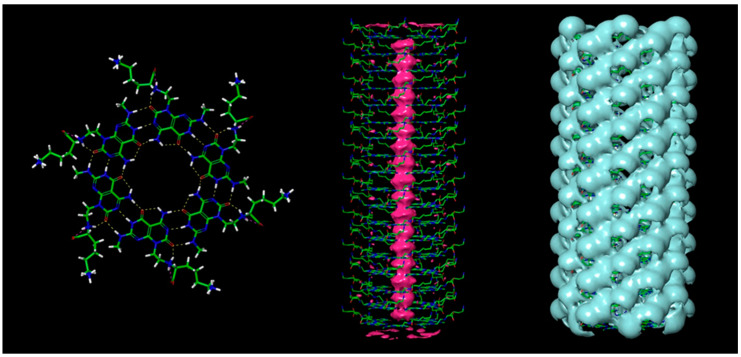
One type of Janus-based nanotubes (JBNTs) developed by the Dr. Yupeng Chen, et al. Image taken with permission.

Increased interest in the use of injectable, biomimetic, and biocompatible nanoparticles for anticancer drug delivery has led to the development of DNA-inspired nanotubes such as Rosette nanotubes (RNTs) that have demonstrated to be effective in regenerative medicine due to their biologically derived structure and chemical properties [48]. Janus based nanotubes (JBNTs), as seen in Figure 2, another type of DNA-inspired nanotube, contained hydrophobic hollow channels inside, allowing drug encapsulation, ensuring the capability to deliver water-insoluble drugs, while allowing prolonged drug releases with the presence of a hydrophilic outer surface. When combined with proteins found in the extracellular matrix (ECM), such as collagen, JBNTs can serve as an adhesion focal point for cells to adhere to. Their nanophase materials have shown great potential to serve as successful implant materials [49]. For drug delivery purposes, RNTs or JBNTs can be processed into non-covalent nanoparticles (such as nanopieces or NPs). The NPs can be used to inhibit disease mRNA or miRNA expression [50,51]. In an anticancer application [51], NPs were shown to deliver oligonucleotides intracellularly to human tumor cells in vitro and in vivo and can inhibit expression of oncogenic miRNA, which in turn can restore the expression of RGS16.

### 2.6. Exosomes Nanoparticles

Recent studies show that exosomes have potential uses in cancer immunotherapy because of their molecular transfer functions and immunogenicity. Moreover, exosomes show their advantages on high biocompatibility, prolonged circulation time, and a high potential for in vivo delivery. However, exosomes require additional steps for antibody encapsulation and complex purification steps, lack of cell specificity, and scalability issues. Proteins can be encapsulated into the lumen of exosomes through genetic engineering of the donor cells or through directly loaded into the exosomes [52]. For example, Yim et al. have shown how to encapsulate the cargo into exosomes by using an optically reversible protein–protein interactions (EXPLORs) approach. This approach conjugated a truncated version of the Arabidopsis CIB1 protein (CIBN) to a cargo protein via light-absorbing photoreceptor crypto chrome 2 (CRY2), which encapsulated the protein inside the engineered exosomes. They demonstrated that EXPLORs treatment on target cells could deliver proteins, such as mCherry, Bax, and IkB protein, and Cre recombinase, which was successfully delivered into parenchymal brain cells in vivo [53].

### 2.7. Protein Nanoparticles

Biopolymer-based nanoparticles, including protein nanoparticles, have been actively developed in the recent decade due to their many desirable properties, such as low toxicity and biodegradability [9]. They offer similar benefits when compared to polysaccharides nanoparticles. Unlike carbohydrates-based nanoparticles, they also offer additional advantages, such as non-antigenicity, metabolizable, and greater stability during in vivo storage. Albumin [54,55], gelatin [56,57], elastin [9,58], and many more are studied extensively for the development of protein-based nanoparticles. For example, Abraxane is used in the current cancer therapeutics. The paclitaxel-bound albumin nanocomplex has a diameter of around 130 nm and is one of the best-selling anticancer drugs in the market for breast cancer [59].

Serum proteins have been explored as naturally derived nanocarriers that are biocompatible and biodegradable, a requirement for cancer nanomedicine. Proteins, made up of amino acids, are one of the most abundant resources in the human body for body maintenance, growth, and repair. They are often used for small-molecular therapeutic drugs and imaging agents due to their minimal toxicity and immunogenicity, which also make them an excellent candidate for vaccine excipients. Although serum proteins are often used to conjugate therapeutic drugs, imaging probes, and targeting ligands, the random distribution of many amine and carboxyl groups in proteins causes irregularity of conjugation with target ligands [59]. The general strategies of developing protein-based nanoparticles may include desolvation, emulsification, biomineralization, and covalent/non-covalent interactions. These protein-based nanoparticles are different from many synthetic polymers due to being metabolizable by digestive enzymes into peptides and can offer several surface modification possibilities due to the presence of functional groups on the surface to enable specific targeting to the site of action [57]. They make a great candidate for drug and gene delivery. Naturally derived proteins, such as albumin, have been used in the market as anticancer treatments [59,60].

Protein-based nanoparticles can be prepared by the self-assembly of protein structures. For example, the E2 component of pyruvate dehydrogenase self-assembled to form a highly thermostable dodecahedral caged structure, which is used for protein-based nanocarriers for lymphatic transport and dendritic cell (DC) uptake. These E2-derived protein nanoparticles showed impressive DC activation by co-delivering a peptide epitope from OVA with the adjuvant using the E2 nanoparticles, which leads to increased and prolonged antigen-specific CD8 + T cell activation [61]. Recently, recombinant protein in designing heat-shock proteins (HSPs) has been served as a carrier for the antigens of interest, loaded through the chaperon-binding properties. Injecting recombinant HSPs nanoparticles containing gp100, MAGE, or HER2 antigens resulted in a higher level of antigen-specific IFN-production, supporting T cell activity. This leads to an increase in survival time for tumor-bearing animals immunized with HSPs-antigen complexes [62].

### 2.8. DNA-Based Nanoparticles

DNA molecules are one of the most fundamental aspects of human life. The development of the novel group of DNA-based therapeutics has included plasmids containing transgenes for gene therapy, oligonucleotides for antisense and anti-gene applications, ribozymes, DNAzymes, aptamers, and small interfering RNAs (siRNAs) [63]. With the ever-growing genomic data collected in the Human Genome Project, potent DNA-based drugs could be developed and help determine genetic markers for drug therapies, interactions, and side effects [64]. DNA molecules can be programmable and synthesized to obtain the desired properties for drug delivery in terms of size, shape, structure, and specific surface functionality [65]. It is also possible in designing DNA nanostructures to target tumor recognition, or have the capability of releasing drugs based on stimuli, making them a highly attractive option as delivery vehicles [66,67].

An injectable, shear-thinning, and DNA-based self-healing hydrogel was developed with oxidized alginate (OA) and silicate nanoparticles for the sustained delivery of simvastatin [68]. They found that OA could create covalent yet reversible imine bonds to interconnect the DNA strands, which proved to show the self-healing properties to the formulated network. The silicate nanoparticles, on the other hand, induce the formation of additional crosslinking points, leading to improvements of shear strength of the hydrogel as well. DNA-based nanoparticles are novel classes of delivery systems for a wide variety of bioactive agents. Plasmid DNA (pDNA) was used as a therapeutic mAb expression platform in vivo by Jacobs et al. [69]. They were able to show that both intramuscular and intratumoral delivery of pDNA showed significant antitumor responses—an alternative approach when conventional administration methods are not enough.

DNA nanostructure can provide drug delivery for cancer therapy. DNA, being a genetic material, has high biocompatibility and low cytotoxicity, which makes it ideal for vectors. Recently, DNA based nanoparticles called DNA nanoclews (DNA NC) can load and deliver Cas9 protein with a sgRNA for genome editing. DNA NC delivered the Cas9/single guide RNA complexes to the nuclei of human cells, allowing targeted gene editing while maintaining cell viability [70]. Additionally, a new kind of DNA nanostructure produced from rolling circle amplification (RCA) exhibited high stability with facile functionalization, properties that are favored in the development of drug delivery platforms. In a paper published by Zhao et al. [71], they introduced an aptamer structure for cancer cell targeting and a hairpin structure for Dox loading and pH-responsive sustained release, which will open a new area of development for functioning DNA nanostructure for chemotherapeutic applications in the future.

### 2.9. Inorganic Nanoparticles

Inorganic nanoparticles have shown promising delivery vectors for delivering intracellular antibodies. There are several types of inorganic nanomaterials, such as gold nanoparticles, carbon nanotubes, quantum dots, calcium phosphate, porous silicon, and mesoporous silica nanoparticles (MSN). The benefits of using these materials include high drug loading, ease of functionalization, a large surface area, and the ability to sustain and control release [72].

Gold nanoparticles (AuNPs) have been used for transporting the proteins. Sokolov et al. has shown an AuNP-based antibody delivery system for intracellular actin targeting and imaging. Anti-actin antibodies were conjugated with the AuNPs and further modified with TAT-HA2. The AuNP-based cytosol antibody delivery system has demonstrated the cytosolic delivery of anti-actin antibodies for therapeutic applications [73]. Gili Bisker et al. utilized the gold nanospheres conjugated to Rituximab, an anti-CD20 monoclonal antibody, and showed proof-of-concept to deliver antibodies with the controlled release by the plasmonic shockwave effect [74]. For the immunostimulatory agent’s delivery, AuNPs were used as substrates and were coated with multilayers to embed antigens [75]. For instance, AuNPs with conjugated tumor-homing peptides encapsulated the TNF and delivered to the CD13 expressed tumor endothelium, releasing its TNF cytokines. Arginine-functionalized gold nanoparticles comprising chemically modified Cas9 protein and sgRNA were used to effectively deliver the Cas9 protein to the cytoplasm of HeLa cells [76]. Cationic gold nanoclusters were assembled with the Cas9 protein and pDNA encoding sgRNA via electrostatic attraction, which targets the polo-like-kinase (Plk1), a master regulator in mitosis, overexpressed in many different types of cancer, and further showed inhibition of tumor growth in mice [77].

Mesoporous silica nanoparticles (MSN) were used to encapsulate the antibody and deliver it to cells. Silica nanoparticles provide several advantages, such as functionalization capabilities and tunable pore size and volume. A study shows that anti-CTLA4 immunoglobulin G (IgG)-loaded MSN delivers the mAb against immunoregulatory molecule CTLA4, which modifies the host response to the tumor leading to antitumor activity [78]. Similarly, porous silicon (pSi) was also used to encapsulate the antibodies in films, membranes, micro, and nanoparticles. Andrew JS et al. reported that bevacizumab was encapsulated into nanostructured mesoporous silica films by electrochemical etching of single crystalline silicon [79]. Kane et al. reported the silica nanoparticle has successfully delivered antibodies into intracellularly. Silica nanoparticles were surface modified with n-octadecyltrimethoxysilane (n-ODMS) and encapsulate with proteins and antibodies [80]. Yuan et al. encapsulated the anti-endothelial growth factor (EGFR) antibody cetuximab in a biodegradable silica nano quencher (BS-qNP). For example, it showed that full-length of IgG molecules and galactosidase were loaded onto amine-functionalized mesoporous silica nanocapsules and delivered into CHO-K1 cells [81]. In the case of adjuvants delivery, the hybrid method of liposome-coated MSNs showed an effective approach. Liposome-coated MSNs were loaded with doxorubicin and oxaliplatin as apoptosis inducers with the indoximod, and an immunometabolic adjuvant the tumor had successfully obstructed the immunosuppressive pathways [82].

A carbon-based nanomaterial has been explored for the intracellular delivery of antibodies. Tumor-specific monoclonal antibodies, fluorescent probe, and radiometal ion chelates have been attached to single-wall carbon nanotubes (SWNTs), which targets the tumor (lymphoma). Multi-walled carbon nanotubes (MWCNTs) have been conjugated with an antigen, tumor lysate protein, to specifically increase the antitumor immune response [83]. Wanichwecharungruang et al. reported oxidized carbon black nanoparticles for intracellular delivery of antibodies. The ability to deliver big therapeutic proteins into cells opens new avenues for antibody therapies [84]. Kam et al. used an SWNT-biotin conjugated with streptavidin resulting in the internalization of the protein into cancer cells [85].

Metal-organic frameworks (MOFs) have been reported to deliver CRISPR/Cas9 components, a subclass of coordination polymers, composed of metal ions that are complexed with organic ligands. The formed MOFs showed a three-dimensional structure with the empty pockets of tunable volumes to host molecular cargo. Recently, zeolitic imidazole framework-8 has shown promising results to deliver Cas9 protein and sgRNA to the ovary cells in a Chinese hamster [86]. Another promising candidate for CRISPR/Cas9 delivery is the NP comprised of dual functionalized graphene oxide (GO), PEG, and PEI. The NPs were formed by modifying planar GO with PEG and conjugated with PEI, further complexed with sgRNA and Cas9 protein. This NP demonstrates the editing of human gastric adenocarcinoma cell line of EGFP [87].

## 3. Cancer Treatments

Nanoparticles as nanocarriers encapsulating chemotherapeutic drugs are one of many available therapeutic treatments in the market, but they are still lacking in targeting efficacy. Therefore, both academia and pharmaceutical industries have focused on delivering active peptides and proteins, a beneficial consequence of the development of biotechnological techniques and genetic engineering [88]. These diverse protein therapeutics, such as human antibodies, chimeric proteins (chimeric antigen receptor (CAR) T cell therapy), and new protein scaffolds capable of binding to “undruggable” targets, provide effective therapies for many human diseases, including diabetes, infection, and inflammatory diseases [89].

**Figure 3:**
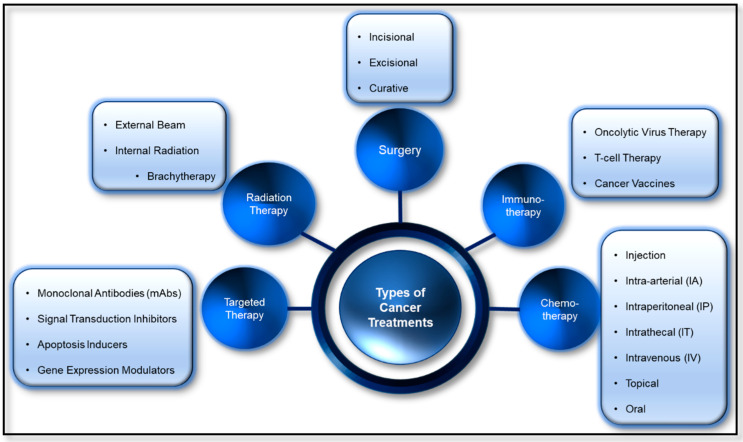
A few types of cancer treatment available for cancer patients.

There are different kinds of cancer treatments available for patients with cancer, as seen in Figure 3. Some may only need one kind of treatment, but others have a combination of treatments, such as surgery, chemotherapy, and immunotherapy, which can help overcome the tumors. Targeted therapy and immunotherapy have shown potential in their application in cancer treatment. Cancer is an uncontrolled growth of abnormal cells in the body. Typically, surgery is one of the most effective therapies for the removal of localized cancers or tumors, however, in cases where cancer becomes metastatic, surgery becomes ineffective, and chemotherapy becomes the most promising therapy to cure cancer in the body, where it could reach every organ via blood circulation [90,91]. Drugs—such as cisplatin, doxorubicin, and paclitaxel—in chemotherapy aid in the inhibition of cell growth in tumor cells also play a major role in deterring other cells from growing, resulting in cell death, leading to severe or life-threatening side effects.

### 3.1. Targeted Therapy

Cancer cells tend to overexpress certain molecules, allowing the high intensity of cell signaling that leads to high survival and division and inhibits cell death/apoptosis. The discovery of cell signaling pathways for proliferation in the early 2000s allowed scientists and researchers to target cells with higher precision than before [92]. However, cancer is a general term for overgrown cells that do not experience cell death, and not all cancer cells are the same. They are normally characterized as having lost their normal cellular regulatory processes, resulting in tumor cells. Tumors cells, such as breast cancers, are not as well infiltrated by the immune system as other cancer types; therefore, the best way to clear these cancers is with targeted therapy [93]. For some types of cancer, only one kind of therapy, such as surgery or radiotherapy, is needed to be effective. However, for some, a more targeted therapy may be necessary for “unapproachable” targets, such as the anti- human epidermal growth factor receptor (HER2) antibody trastuzumab (Herceptin), targeting the HER2-positive breast cancer [93]. Human epidermal growth factor receptor 2 (HER2) is a gene for a protein that has a HER2 receptor, which is responsible for how a healthy cell grows, divides, and repairs itself. When there is an overexpression of the HER2 receptor, it causes cancer to develop, divide, and multiply in an uncontrolled way. People with HER2 positive cancer have too many HER2 genes and may trigger cells to proliferate quickly [94]. Trastuzumab (Herceptin) is a monoclonal antibody, a type of targeted therapy anticancer drug and a biological drug, used to treat breast cancer and stomach cancer, targeting HER2 positive receptors found in tumor cells [94,95,96].

Many cancer drugs are considered undruggable due to the lack of target-specificity. For example, cancer chemotherapeutic drugs, such as targeting DNA synthesis (nucleoside analogues), targeting DNA (alkylating agents), microtubules (vincristine), and anthracyclines (directed at various cellular targets) show remarkable response, but come with major side effects [97]. Antibodies are also widely used to target externalized antigens, engineered to enter cells, or are expressed intracellularly with the aim of intracellular binding antigens. Specifically, monoclonal antibodies have been successfully used to combat many cancers and autoimmune diseases [98]. Moreover, there are various molecular activities leading to pathology, which can be attractive targets for biopharmaceutical development. For example, tumorigenesis, such as c-myc, Kras, and fusion transcription factors related to pediatric cancers can be a potential target site for monoclonal antibody treatment. Currently, most applications are focused on targeting proteins related to oncological malignancies. Antibodies can target virtually any disease that involves intracellular components. Intracellular antigens can become externalized on the cell membrane surface or secreted and can, therefore, be targeted by antibodies [97].

### 3.2. Immunotherapy

Immunotherapy is a type of biological therapy for cancer treatment that uses the patient’s own immune system to fight cancer. One of the purposes of the immune system is to detect and destroy abnormal cells and decrease the growth of many cancer cells from dividing and multiplying. Immunotherapy uses different ways to boost the body’s immune system to kill cancer cells, such as nonspecific immune stimulation, T cell transfer therapy, and immune checkpoint inhibitors. While nonspecific immune stimulation therapy stimulates the patients’ immune systems in a general way, T cells are taken from patients and engineered to be more efficient in killing cancer cells in the lab before returning them to the patients to be used as cancer therapeutic treatments. On the other hand, immune checkpoints on cancer cell surfaces keep the T cells from turning on and killing them. Therefore, immune checkpoint inhibitors were developed to block the checkpoints to allow the T cells to attack cancer cells [99].

Immunostimulatory agents, such as adjuvants, cytokines, and monoclonal antibodies hold great potential for cancer treatment. These antigenic materials can generate potent tumor-specific responses and have been investigated as monotherapies for nonspecifically boosting immune activity. The specific immune responses to cancer depend on antigen uptake by antigen-presenting cells (APCs), particularly dendritic cells (DCs) [100]. For example, immunostimulatory agents, such as adjuvants, can be loaded with antigenic material into nanoparticle systems to enable delivery to specific immune cell subsets, such as antigen-presenting cells (APCs). This leads to stimulating the immune process, such as T cell stimulation, and generates antitumor responses [101].

### 3.3. Combination Therapies

In 2011, anti-CTLA4 therapy (ipilimumab) was approved for late-stage melanoma and the success of this immunotherapy, in general, has become the motivation behind many combination therapies, with other conventional therapies to improve the effectiveness of cancer treatment [102]. It was observed that a combination of radiation and immunotherapy is beneficial for various cancer types [103,104,105]. During the development of tumor cells, the relationship between the tumor and immune system would start from tumor cells being recognized and destroyed (immune elimination) to coexisting together (immune equilibrium), and finally immune escape, where the immune cells do not recognize the tumor cells as abnormal. In this stage, there is a poor antigen presentation masking tumor from immune detection and elimination. Radiation therapy can downregulate the inhibitory ligands and cytokines, increase MHC class I expression, and, therefore, make it visible to both types of immune systems. After the “unmasking” of antigens in the tumor cells, the chimeric antigen receptor (CAR) T cell therapies can be applied to strengthen the host’s immune system and eliminate cancer cells more efficiently in the body [102].

Proteins as therapeutic agents for cancer treatments make protein delivery an important area of research. Monoclonal antibodies, hormones, and vaccines have played an essential part in preventing and impeding more than 100 diseases, including cancer, infectious disease, autoimmune diseases, HIV/AIDS, and many more [106]. There is no doubt that targeted therapy and immunotherapy for cancer treatment will continue to thrive due to their increased effectiveness with fewer side effects when compared to radiotherapy or chemotherapy. Many diseases have shown to arise from the dysfunction of intracellular proteins; therefore, multiple efforts were made to understand the mechanism behind intracellular delivery of proteins into cells and applying these understandings into making more efficient treatments than the previous generations. However, delivering proteins or peptides has been challenging because of their large sizes, short in vivo half-lives, high elimination rates, limited abilities to cross cell membranes, and poor bioavailability [106,107]. Therefore, this review focuses on the mechanism of intracellular delivery of proteins, such as antibodies, antigens, and gene editing proteins into host cells, to achieve anticancer effects that overcome current obstacles, as well as the applications of protein delivery in anticancer treatment.

## 4. Proteins and Peptides Delivery as Anticancer Therapeutics

Developments in cancer nanomedicine in the past decade have focused on enhancing efficacy while reducing adverse side effects. Researchers often focus on the ability of the delivery vehicles or the drugs themselves to target tumors and improve the biodistribution in the body [59]. The rest of this review discusses the different biomaterials, especially nanoparticle-based therapeutic strategies, ranging from the use of protein—based as naturally derived nanocarriers for anticancer drugs—to exploiting the function of T cells by developing antigen/adjuvant vaccines, or genetically engineered T cells to improve the immunity of the hosts, and gene modification—especially using CRISPR/Cas9 system.

### 4.1. Vaccines/Adjuvants

Vaccines have been viewed as one of the most successful health measures of all time in preventing infectious diseases caused by viruses [108]. Certain cancers, such as human papillomavirus (HPV), are caused by viruses and, therefore, vaccines were developed to help protect against infection caused by the viruses [109]. An adjuvant is a substance included in a vaccine that stimulates the immune system by activating pathways that the hosts naturally use to recognize bacteria or viruses. In adjuvant vaccines, the adjuvant is carefully selected combine with a specific antigen to generate specific and long-lasting immunity against the target antigen [110,111,112]. The development of nanoparticles encapsulating specific antigens can be effective as adjuvants because they provide sustained release of the antigen [113]. Cage protein (CP) nanoparticles are self-assembled protein structures that attract attention as a vaccine platform and have the potential to improve vaccine efficacy by promoting antigen localization and enhancing endocytosis of antigens by antigen-presenting cells (APCs). Unlike virus-like nanoparticles (VLPs), CP nanoparticles do not come from viral sources, even though they have virus-like structures and geometries [61]. Immunotherapy utilizing vaccines have been one of the many cancer therapeutic treatments based on tumor-associated antigens (TAA) inducing specific and desired T cell responses without harming normal cells [112].

T cells play a central role in the immune response. They originate from hematopoietic stem cells, produced from the bone marrow. Each cell develops its own T cell receptor (TCR) that is specific for a particular antigen, maturing in the thymus, and leaving to circulate through the peripheral lymphoid organs, ready to encounter specific genes and be activated [114]. T cell lymphocytes, especially cytotoxic T cell lymphocytes (CTL), play an essential role in recognizing and killing their targets once the antigens are detected, producing cytokines, and regulating the immune response. Hence, many researchers have been taking advantage of their roles as cell killers to develop macro- and nanoparticles to be used as the antigen/drug delivery system. When delivered into the body, the cargo, usually drugs, is protected from degradation, therefore increasing the half-life of the encapsulated antigen and the immunomodulators in vivo [115]. These particles are specifically engineered to target specific cell types, reducing off-target side effects. An example is demonstrated by the Serda group [116] where they showed PLGA encapsulated antigen-induced T cell response with a 1000-fold lower dose compared to the nonencapsulated antigen. Different vaccine particle sizes may go through different uptake routes as described in Khong et al. in the paper published in 2016 [115]. While the complex fabrication process to develop natural proteins as nanocarriers or drugs is still impeding the use of these nanocarriers, it is worth noting that they may greatly improve the response rate and the potential advantages they possess may be the motivation behind many investigations of these nanocarriers [59].

Although vaccinations can enhance tumor-specific immunity, it was observed that many patients do not seem to respond due to the lack of tumor-reactive T cells or poor T cell infiltration to the tumor. The need for combining different adjuvants into a single vaccine may be the next logical step to increase vaccine efficiency. It was demonstrated that various adjuvants come with both desired and undesired traits. It is hopeful that, by combining different adjuvants, the immune response can be skewed toward the favorable one. Combination therapies may be possible, where the synergy between peptide vaccines and checkpoint inhibitors can be obtained [115], with a note given to not induce excessive T cell sequestration. Implantable and integrated smart release drug delivery and sensor devices were proposed for the distribution of insulin via feedback-controlled systems [115].

### 4.2. Monoclonal Antibodies (MAbs)

Another immunotherapeutic treatment using monoclonal antibodies (mAbs) is called adoptive cell transfer (ACT). Patient T cells grown in the lab can recognize and kill cancer cells more efficiently compared to native immune cells. The process involves specific tumor-associated antigens (TAAs) being recognized by the genetically modified T cell, more commonly known as chimeric antigen receptors (CARs). In 2017, only two CAR T cell immunotherapies were approved by the Food and Drug Administration (FDA), one for children with acute lymphoblastic leukemia (ALL) and another for adults with advanced-stage lymphomas [117]. Initial T cell developments start with using the patient’s own cells, which are collected and genetically modified via ex-vivo transduction using a product-specific vector. After several cell expansions, with carefully controlled manufacturing processes, they are then administered back to the patient. The personalized, specificity, and selectivity receptors minimize off-target toxicity and enhance antitumor efficacy [118]. However, the problem occurs when the patients themselves are not healthy; therefore, impeding the proliferation rate of the cells, resulting in suboptimal cell numbers for some patients. To adapt to the fast-evolving and novel technologies, genetic engineering is utilized, by manipulating genetic codes of cells to attain better specificity and selectivity in detecting and destroying cancer cells.

### 4.3. Genetic Engineering—CRISPR/Cas

**Figure 4:**
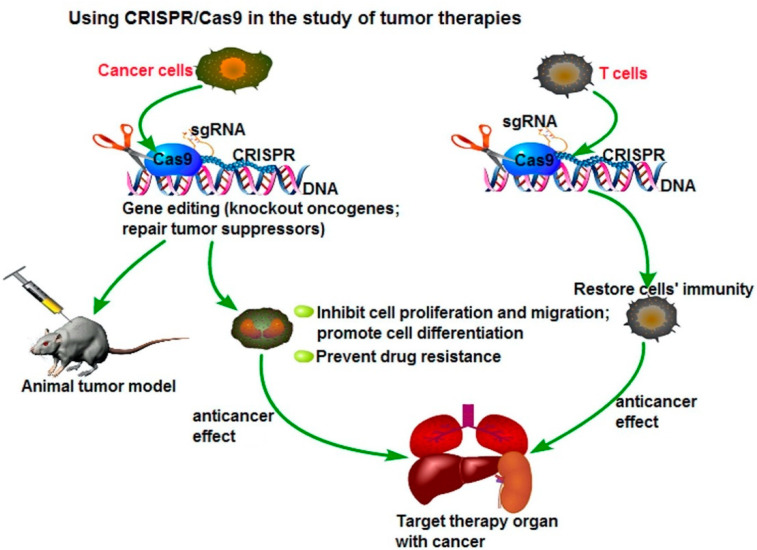
Using CRISPR/Cas9 in the study for tumor therapies. Reprinted with permission Figure [124], Clinical Genetics, 2019.

Although gene modification approaches have gone on for many years, its limitations, such as low efficiency of gene targeting, remain one of the challenges that researchers are currently facing. One strategy that has been studied to increase efficiency is to introduce the DNA double-strand breaks (DSBs) at the genomic locus of interest, interest [119], resulting in the development of site-specific nucleases, such as zinc-finger nucleases (ZFNs) [120,121,122,123]. However, CRISPR-Cas9 methods are cheaper, faster, and more accurate because they only require a short stretch of RNA to target a genomic site. This method can easily and selectively disable or change genes in human cells, providing promising gene therapy treatments to cancer and inherited genetic disorders. A possible application of the CRISPR/Cas9 system to cancer treatment is to directly target the tumor marker in cancer cells and eliminate the genetic alterations of tumor proliferation and metastatic capacity, as seen in Figure 4. Another potential therapeutic application could be the fusion of dead Cas9 (dCas9) to histone modifiers and proteins involving altering DNA methylation, to target cancer epigenetics, such as acute lymphoblastic leukemia or Ewing sarcoma. The delivery of Cas9 protein with sgRNA is the most straightforward way to achieve gene editing, leading to minimal off-target effects and toxicity due to the transient functionality [124,125].

The CRISPR/Cas9 system, currently one of the more commonly known genetic engineering methods, is an adaptive immune defense mechanism, which is a naturally occurring DNA-cutting system found in bacteria for degradation of foreign genetic material [126]. In a paper published in *Science* in 2012, it was demonstrated that this genetic scissors can be used as a genome-editing tool [127]. CRISPR stands for clustered regularly interspaced short palindromic repeats and this versatile system consists of two biological components, the CRISPR RNA (crRNA), and the CRISPR-associated endonuclease (Cas9) modules. In general, the endonucleases break the double-stranded DNA and crRNA will then target the specific DNA sequence. The single guided DNA (sgDNA) produced from the crRNA module, and Cas9 can be optimized without altering the function of each other. In mammalian cells, gene knockout is highly prevalent when Cas9 is targeted to the exon regions of specific genes [120]. CRISPR–Cas9 becomes useful when one can identify a target gene or protein during the drug discovery process. CRISPR–Cas9 allows the editing of genes that cause cancer cell proliferation; it can be used to insert suitable genes after the “cut”. Since its discovery, many researchers have started exploring the roles of genes implicated in cancer initiation, progression, and therapeutic response [119] and apply CRISPR/Cas9 in their investigations.

The strategies for delivering CRISPR-Cas9 via delivery vehicles are often impeded by the high molecular weight and complexity of the system. However, as mentioned previously, researchers developed new nanoclews, a vehicle covered with positively charged material, such as lipids, which can disrupt the endosomal membrane but remain free inside the cell [128]. CRISPR–Cas9 systems have also shown multiple advantages over many conventional gene technologies, where researchers can develop genetically modified T cells that enhance their ability to detect and kill cancer cells. The T cells can be modified in a way where they express the chimeric antigen receptors (CARs) on the surfaces, enabling the detection and destruction of specific cancer cells [129]. Researchers in Sichuan University in China injected a non-small cell lung cancer (NSCLC) patient with genetically modified T cells containing the CRISPR-edited gene in October 2016 [130,131,132]. PD-1 genes, commonly found in NSCLC patients, are an “off-switch” for T cells from damaging healthy tissues, but cancer cells can hijack the system and avoid detection from T cells. Therefore, genetically modified PD-1 knockout T cells are injected back into patients, and subsequently are able to identify and attack the cancer cells. Similar clinical trials using CRISPR as cancer immunotherapy are also ongoing in China for esophageal cancer patients, as well as patients with B-cell lymphoma and leukemia [133,134].

## 5. Current Applications and Ongoing Clinical Trials

Protein or peptides as therapeutic cancer agents were studied extensively using cytokines, antibodies, enzymes, tumor antigens, pro-apoptotic protein/peptides, and others [135]. The use of interleukins (ILs), interferons (INFs), and tumor necrosis factors (TNFs) are example of cytokines used for cancer therapy to induce tumor cell apoptosis or regulating responses [136]. Antibodies have been used to specifically target oncogenic proteins and are shown to be one of the most successful approaches for cancer treatment [136,137]. As mentioned previously, mAbs against different cancers, such as HER2, or vascular endothelial growth factor (EGFR), are used to achieve remarkable antitumor activity [137].

One of the current applications that uses nanotechnology in cancer treatments is the development of FDA-approved nanoparticle-bound albumin for breast cancer, pancreatic cancer, and NSCLC. Although it has been approved for use since 2005, researchers, and clinicians have not stopped looking for ways to alleviate the experience by patients better when taking the drugs. Many liposomes were used as drug delivery vehicles for different diseases, such as the treatment of Kaposi’s sarcoma via doxorubicin encapsulated in liposomes and vincristine encapsulated in liposome for acute lymphoid leukemia [138]. In addition to the use of paclitaxel as a cancer drug therapy, many nanotechnology uses are still in the developmental phases. When paclitaxel was first used, it was synthesized with solvent bound cremophor, but it caused many adverse events such as neurotoxicity [139,140]. To reduce neurotoxicity and hypersensitivity of the drug, different formulations for delivery of paclitaxel, albumin-bound paclitaxel, Abraxene^®^, was developed. It was also found that administering albumin-bound paclitaxel over a short period of time, when compared among 1 h versus 3 h or 3 h versus 24 h, generally increased neuropathy adverse events. Many uses of albumin-bound paclitaxel in clinical studies were only evaluated for 30 min infusions, which could be the cause of the increased numbers of neuropathy, when observed. A 2 h infusion of albumin-bound paclitaxel, Abraxene^®^, was used in the Phase 2 clinical trial led by Paik et al. in 2011 [140]. Although there was a significant decrease in average peripheral neuropathy experienced by patients, it did not affect the survivability of patients or elevate the other adverse events. The data suggests that changing the infusion time from 30-min to 2 h may lead to significant reduction in adverse events, but did not mention the significance of the use of Abraxene^®^ in the study. It is also important to note that the study conducted by Paik et al. in 2011 was supported by Abraxis BioScience.

Combination therapy studies, such as combining CRLX101, a nanoparticle camptothecin (CPT), with enzalutamide, in people with abnormal growth of prostate cancer—even at low testosterone levels in the body—are currently being conducted [141]. Enzalutamide is a modern hormonal treatment for castrate-resistant prostate cancer, but it only works for a certain amount of time before the cancer becomes resistant to it. CRLX101, on the other hand, is an investigational polymeric nanoparticle-drug conjugate containing CPT, which has a preference to accumulate in tumors through the enhanced permeability and retention (EPR) effect [142,143], and has a long circulation time [144]. CRLX101 can increase therapeutic efficacy in vivo by inhibiting DNA repair and hypoxia-inducible factor 1 alpha (HIF1 α), pathway activation in tumors. Therefore, researchers are investigating the combination therapy of CRLX101 with enzalutamide to combat this disease. The study started in March 2019, and is currently ongoing, with an estimated completion date of June 2021.

CRLX101 is a polymeric nanoparticle comprised of the linear cyclodextrin-polyethylene glycol (CD-PEG) co-polymer as the backbone, covalently conjugated with the drug, CPT (Cheng J et al., 2003 Synthesis of Linear beta-cyclodextrin and Cheng J. 2004, antitumor activity of beta-cyclodextrin). CRLX101 was shown to improve the efficacy of the drug itself by increasing intracellular CPT deposition and providing a sustained supply of CPT, which is highly insoluble in water (approximately 4 µg/mL) and highly unstable, being susceptible to spontaneous and reversible hydrolysis, resulting in an inactive carboxylate form in physiologic pH [145,146]. This inactive form of CPT has a 200-fold greater affinity to human albumin serum than the active form; it has shown high drug-related toxicity by limiting antitumor efficacy. CD-PEG polymers are highly producible with diameters between 30 and 40 nm [147]. The addition of the drug, CPT, results in a neutral surface charge and the PEG blocks improved solubility and stealth properties, which minimizes immunogenicity as well. The studies confirmed that CPT is linked through glycine to form ester linkage for covalent attachment to CD-PEG and that it successfully stabilizes the labile lactone ring of CPT, preventing premature CPT inactivation; therefore, increasing an active form released in a controlled manner and over a sustained period. While CRLX101 showed significant promises on clinical outcomes and seems to be a highly versatile nanotechnology platform, it is important that the activity of the nanoparticles is continuously observed and investigated to ensure the establishment of CRLX101 as a new oncology agent [148].

A Phase 1b clinical trial is testing the safety of an investigational drug PVX-410 combined with pembrolizumab as a treatment for metastatic triple negative breast cancer (TNBC), HLA-A2 + [149]. Pembrolizumab (Keytruda) is a monoclonal antibody checkpoint inhibitor used for a wide range of cancer diseases and is often given when cancer has spread to other parts. The cancer medicine is designed to target and block the programmed death receptor 1 (PD-1) protein on the surface of T cells triggering them to find and kill cancer cells [150]. PVX-410 is a vaccine composed of four 9-amino acid peptides (multi-peptide) that may help the patient immune systems by stimulating the patient’s cytotoxic T-lymphocytes (CTLs) to target specific tumor associated antigens. The vaccine is composed of four different peptides, which are high in selectivity, targeting overexpression of XBP1, CD138, and CS1 [151]. While it has the potential to be utilized as a stand-alone therapy, it is currently mainly investigated with a combination of other cancer drugs. Currently, there are different ongoing clinical trials that involve combination therapies between PVX-410 and different cancer drugs, such as lenalidomide, hiltonol, and citarinostat (CC-96241) [152] to treat different cancers, such as smoldering multiple myeloma, and several solid cancer tumors, including pancreatic and prostate.

An increasing number of protein drugs are in the developmental phases, and some are being discovered that kill tumor cells via different mechanisms. Some protein drugs, such as saporin, which is a 30-kDa, positively charged, and membrane-impermeable ribosome inactivating protein (RIP), can irreversibly inhibit protein synthesis in eukaryotic cells [153]. Despite the different protein therapeutics being developed for the treatment of cancer, further advances are necessary to translate this fundamental research into preclinical or clinical investigation before entering the market. While it is important to search for personalized therapeutic treatments for each patient, a universal, yet potent and stable protein delivery system is needed.

## 6. Future Prospects and Conclusions

In summary, we reviewed the nanomaterials for drug and protein delivery in anticancer applications. We introduced different types of nanoparticles for protein delivery, such as lipid nanoparticles, viral vectors, polymeric, hydrogel, biomimetic, and inorganic nanoparticles. Each nanomaterial offers different advantages and limitations. For example, the polymeric nanoparticle is accessible because of its simple formulation, but suffers from cytotoxicity. Lipid nanoparticles show high biocompatibility, but low effective encapsulation [90,154,155], while antibodies play an essential role in cancer treatment due to their accessible protein expression with high affinity and specificity (although their production is still a significant issue). Most therapeutic applications remain in the in vitro stages, and are rarely applied to in vivo studies, resulting in considerable room for progress. We hope that the development of targeted intracellular protein delivery will treat clinical therapy in the future [156].

The use of nanoparticles as delivery systems for antibodies have great application for cancer treatment, especially in immunotherapy. However, some studies have found that they also impact the immune system (e.g., a cytokine storm). Therefore, further understanding of the mechanisms and influences of nanoparticles on the immune system is strongly recommended for further advancement in nanotechnology [7]. Biomimetic delivery approaches can be a potential solution. For instance, self-assembling peptides are currently emerging as a new trend (as adjuvant-free vaccines). These peptides are synthesized to have domains that help them self-assemble into nanofiber structures [157]. Although the emerging biomimetic delivery discussed has shown significant potential, there is still some room to improve (e.g., enhancing immunostimulatory potency) [158]. The development of novel DNA-based nanotherapeutics, such as Janus-based nanotubes, were proven to be successful and effective in cell penetration in vivo in multiple studies [12,49,159,160]. They can be applied into the delivery of chemotherapeutic drugs, photosensitizers, imaging probes, small interfering RNA (siRNA), and other therapeutic agents due to their highly desired cellular uptake and pharmacokinetics, when compared to synthetic polymers, such as the used of DNA-inspired nanoparticles (e.g., the nanopieces) [160].

The application of the CRISPR/Cas9 system for cancer treatment has sharply accelerated. We discussed several nanomaterials that were reported for in vitro Cas9 RNP delivery into cells, including cationic lipid nanoparticles, DNA nanoclews, gold nanoparticles, and zeolitic imidazole. As different nanocarriers are proposed and developed, the interaction between tumor cells and nanoparticles are being studied. However, many nanomaterials cannot currently be accessed due to the lack of nanomaterials that can effectively deliver Cas9 proteins. One solution for this is using multi-cellular spheroids for the in vitro model, which offers an excellent reference model to assess the efficacy of new nanomaterial formulation [66]. Intelligent vehicle designs could possible overcome the obstacles mentioned, but may inevitably increase complexity of synthesis or formulation, as well as the cost of production. The coalescence among encapsulating therapeutic proteins with the use of genetic engineering, as well as implantable or integrated smart devices for drug delivery, are promising strategies for cancer treatment.

## Data Availability

Not applicable.
